# A highly sensitive novel immunoassay specifically detects low levels of soluble Aβ oligomers in human cerebrospinal fluid

**DOI:** 10.1186/s13195-015-0100-y

**Published:** 2015-03-22

**Authors:** Ting Yang, Tiernan T O’Malley, Daniel Kanmert, Jasna Jerecic, Lynn R Zieske, Henrik Zetterberg, Bradley T Hyman, Dominic M Walsh, Dennis J Selkoe

**Affiliations:** Ann Romney Center for Neurologic Diseases, Brigham and Women’s Hospital and Harvard Medical School Boston, 77 Avenue Louis Pasteur, Harvard Institute Medical, Room 730, Boston, MA 02115 USA; Acumen Pharmaceuticals, Inc., 9816 Easton Drive, Beverly Hills, CA 90210 USA; Singulex, Inc., 1701 Harbor Bay Parkway, Suite 200, Alameda, CA USA; Department of Psychiatry and Neurochemistry, Clinical Neurochemistry Laboratory, Institute of Neuroscience and Physiology, the Sahlgrenska Academy at the University of Gothenburg, S-431 80 Mölndal, Sweden; UCL Institute of Neurology, Queen Square, London, WC1N 3BG UK; Massachusetts General Hospital, Charlestown, MA 02129 USA

## Abstract

**Introduction:**

Amyloid β-protein oligomers play a key role in Alzheimer’s disease (AD), but well-validated assays that routinely detect them in cerebrospinal fluid (CSF) are just emerging. We sought to confirm and extend a recent study using the Singulex Erenna platform that reported increased mean CSF oligomer levels in AD.

**Methods:**

We tested four antibody pairs and chose one pair that was particularly sensitive, using 1C22, our new oligomer-selective monoclonal antibody, for capture. We applied this new assay to extracts of human brain and CSF.

**Results:**

A combination of 1C22 for capture and 3D6 for detection yielded an Erenna immunoassay with a lower limit of quantification of approximately 0.15 pg/ml that was highly selective for oligomers over monomers and detected a wide size-range of oligomers. Most CSFs we tested had detectable oligomer levels but with a large overlap between AD and controls and a trend for higher mean levels in mild cognitive impairment (MCI) than controls.

**Conclusion:**

Aβ oligomers are detectable in most human CSFs, but AD and controls overlap. MCI CSFs may have a modest elevation in mean value by this assay.

**Electronic supplementary material:**

The online version of this article (doi:10.1186/s13195-015-0100-y) contains supplementary material, which is available to authorized users.

## Introduction

Progress in defining the molecular changes underlying Alzheimer’s disease (AD) has led to a modification of the original amyloid hypothesis to postulate that diffusible assemblies (oligomers) of amyloid-beta (Aβ) protein are the principal neurotoxic species and exist in a complex equilibrium with fibrillar amyloid plaques [1]. A problem in advancing this Aβ oligomer hypothesis has been the difficulty in pinpointing the sizes and levels of the major bioactive oligomers. A bewildering array of oligomeric species has been described, almost entirely based on studies of synthetic Aβ peptides of defined lengths used at supraphysiological concentrations [2]. Going forward, the field should focus on characterizing and quantifying natural Aβ oligomers (oAβ) isolated from human brain tissue and biological fluids.

While natural oAβ can be readily identified in aqueous extracts of AD brain tissue, attempts to detect soluble oAβ in cerebrospinal fluid (CSF) have led to mixed results, with a few laboratories reporting their ready detection but others concluding that their levels are very low or virtually undetectable (discussed in [3]). In the past 18 months, at least five studies reported measuring oAβ in CSF from AD and control patients [4-8]. All five found considerable overlap between the levels of oligomers detected in AD and control CSF, but four studies reported higher mean levels of soluble oligomers in AD than control subjects. One study examined only postmortem CSF [7]. Two of the studies reported that measurement of soluble oligomers was less good at discriminating AD from controls than were Aβ42 or tau levels [5,6]. Interestingly, Holtta and colleagues found that AD patients with mild and moderate AD had significantly higher mean levels of oAβ than controls, whereas patients with severe AD did not differ significantly from the control group [5]. Most of the groups describing positive detection of natural oligomers did not report the rigorous characterization of the assays; for example, by serial dilutions to confirm the linearity of the signal, by immunodepletion to confirm their Aβ specificity, by appropriately determining the lower limit of quantification, by excluding spurious signals arising from heterophilic antibodies, and by recovery of signal from samples spiked with an oligomer standard.

Two recent examples of detailed studies that were unable to establish the existence of oAβ in human CSF despite the use of well-characterized immunoassays were those by Esparza and colleagues [9] and by Yang and coworkers [3]. In the latter report, our group postulated very low or virtually undetectable levels of oAβ in the CSF, and we then provided evidence that the relative hydrophobicity of oligomers could lead to very brief dwell times in aqueous compartments such as CSF or interstitial fluid [10].

To quantify the very low CSF levels of soluble oligomers and show diagnostically meaningful differences between AD and normal subjects will require highly sensitive assays that specifically detect oligomers and are blind to the abundant Aβ monomers present in all CSF samples. In this regard, we were intrigued by a very recent report of successful quantification of soluble oAβ by an immunoassay that utilized the high-sensitivity Singulex Erenna platform (Singulex, Alameda, CA, USA) [8]. The authors even reported observing modestly but significantly higher levels of the oligomers in AD CSF compared with age-matched controls. Accordingly, we decided to try this platform first using a new oAβ-specific monoclonal antibody we generated and then employing the antibodies with the same complementary determining regions as those used by Savage and colleagues [8]. Here, we report achieving a similar degree of sensitivity and specificity with our new sandwich immunoassay, but the signals in two cohorts of AD and age-matched control CSF samples overlapped substantially, while mild cognitive impairment (MCI) subjects showed a trend toward having slightly higher levels. The implications of this new work with regard to the usefulness of oligomers as an AD biomarker and the development of different and even more sensitive Aβ oligomer assays are discussed.

## Methods

### Antibodies

Monoclonal antibody (mAb) 3D6 is highly specific for the extreme N-terminus of Aβ [11] and was kindly provided by Dr Guriq Basi and Dr Dale Schenk (Elan, plc, South San Francisco, CA, USA). mAb 3B3 was generated by immunizing mice with an Aβ1–42 preparation known as amyloid-beta-derived diffusible ligand (ADDL) and was provided by Dr Bill Goure (Acumen Inc., Beverly Hills, CA, USA) [8]. 3B3 is the murine precursor of the humanized 19.3 mAb used by Savage and colleagues and has the same complementary determining regions, and it demonstrates similar relative preferences to oAβ as assessed by surface plasmon resonance and KinExA (Sapidyne Instruments Inc., Boise, ID, USA). In solution, our novel mAb 1C22 only weakly recognizes Aβ monomer and binds strongly to Aβ aggregates, but in solid phase and tissue sections 1C22 can also bind to fibrils and amyloid plaques [12]. A commercial N-terminal antibody similar to 3D6, 82E1 [3] was obtained from Immuno-Biological Labs, Inc. (Minneapolis, MN, USA).

### Preparation of synthetic Aβ standards: ADDLs, S26C dimers, and dityrosine dimers

Synthetic Aβ(1–42), Aβ(1–40) and Aβ(1–40)S26C were synthesized and purified using reverse-phase high performance liquid chromatography by Dr James Elliott at Yale University (New Haven, CT, USA), and the mass and purity of peptides were confirmed by liquid chromatography–mass spectrometry.

ADDLs were prepared essentially as we have described previously [13]. Briefly, Aβ(1–42) peptide was dissolved in hexafluoroisopropanol to a final concentration of 1 mM and the solution was incubated at 37°C for 1 hour and mixed briefly by vortexing every 10 minutes. After 1 hour the hexafluoroisopropanol was evaporated using a speedvac. The dried peptide film was stored over desiccant at −20°C overnight. The film was then dissolved in anhydrous dimethylsulfoxide (Life technologies, Eugene, OR, USA) to produce a solution of 22.5 mg/ml and then diluted 1:50 with Hams F-12 media (Life technologies). The resulting solution was incubated at 4°C for ~14 hours and then centrifuged at 16,000 × *g* for 10 minutes. The supernatant was recovered and absorbance at 275 nm recorded using a spectrophotometer with a 1 cm path length microcuvette. The concentration of oAβ was determined using the extinction coefficient, ε_275_ = 1,361/M/cm [14], and by measuring the percent contribution of oAβ using analytical size exclusion chromatography (SEC) (Additional file 1). Relative oligomer content was estimated by dividing the peak area of the oligomer peak by the peak area of the oligomer peak plus the monomer peak. This value (based on three separate experiments) revealed that Aβ species larger than monomers accounted for 47 ± 6% of the total Aβ present in the ADDL preparations used. Following concentration determination, ADDLs were aliquoted (5 μl), immediately frozen on dry ice and then stored at −80°C. Once thawed, aliquots were used immediately either for characterization (using analytical SEC and electron microscopy [14]) or as standards in the MSD Multi-Array (Meso Scale Discovery, Gaithersburg, MD, USA) or Erenna immunoassay system (Singulex).

Monomeric Aβ was prepared as described previously [15]. Briefly, Aβ(1–40) was dissolved at 2 mg/ml in 50 mM Tris–HCl, pH 8.5, containing 7 M guanidinium HCl and 5 mM ethylenediamine tetraacetic acid, and incubated at room temperature overnight. The sample was then centrifuged at 16,000 × *g* for 30 minutes and the upper 90% of supernatant applied to a Superdex 75 10/300 size exclusion column (GE Healthcare Biosciences, Pittsburgh, PA, USA), eluted at 0.5 ml/minute with 50 mM ammonium bicarbonate, pH 8.5, and absorbance monitored at 280 nm. Fractions of 0.5 ml were collected. The UV absorbance at 275 nm was determined for the peak fraction and the concentration of Aβ determined using ε_275_ = 1,361/M/cm [14]. Dityrosine cross-linked Aβ dimer ((Aβ(1–40))_DiY_) was prepared as reported previously [14] and SEC-isolated as described above. The UV absorbance at 283 nm of the dimer peak fraction was measured and the concentration of DiY dimer determined using ε_283_ = 6,244/M/cm [14]. (Aβ(1–40)S26C)_2_ was prepared as described previously [14] and the dimer was SEC-isolated as outlined above. The UV absorbance at 275 nm of the (Aβ(1–40)S26C)_2_ peak was measured and the concentration of this dimer determined using ε_275_ = 2,722/M/cm [14].

Following collection and concentration determination, monomer and dimer peak fractions of the respective oligomer preparations were diluted to 48.5 μM and aliquots (20 μl) were immediately frozen on dry ice and then stored at −80°C. Once thawed, aliquots were used immediately either for characterization (using analytical SEC and electron microscopy [14]) or as standards in the MSD or Erenna immunoassays.

### MSD Aβ oligomer immunoassay

Sandwich assays were performed using 96-well plates and an MSD Multi-Array (Meso Scale Discovery) [3]. mAb 1C22 or 3B3 was used for capture, and biotinylated 3D6 or 82E1 for detection. Samples, standards and blanks were loaded in duplicate, and bound biotinylated mAb measured using SULFO-TAG streptavidin (Meso Scale Discovery). Light emitted from SULFO-TAG at the electrode surface was quantified with an imager (Sector Imager 2400A; Meso Scale Discovery). The limit of detection (LOD) is defined as:

$$ \mathrm{L}\mathrm{O}\mathrm{D} = 2 \times \mathrm{standard}\ \mathrm{deviation}\ \mathrm{background}\ /\ \mathrm{slope}\ \mathrm{of}\ \mathrm{the}\ \mathrm{standard}\ \mathrm{curve}. $$

The lower limit of reliable quantification (LLoQ) is defined as the lowest back interpolated standard that provides a signal two-fold the background with a percentage coefficient of variance (CV) ≤20%.

### Erenna Aβ oligomer immunoassay

The Erenna Immunoassay System (Singulex) is based on single molecule counting technology [16] and typically allows a 20-fold to 100-fold increase in sensitivity compared with traditional detection systems. Most experiments employed our mAb 1C22 for capture and 3D6 for detection, but for one set of experiments 3B3 was used for capture and 82E1 for detection. Biotinylated capture mAbs were bound to microparticles (MPs) at a ratio of 12.5 μg biotinylated antibody per milligram of MPs using a kit from Singulex. MPs with bound capture mAb were diluted to 50 μg/ml in assay buffer (Tris buffer; 50 mM Tris, 150 mM NaCl, pH 7.6), with 1% Triton X-100, 0.0005% (w/v) d-desthiobiotin and 0.1% bovine serum albumin, and 100 μl of this suspension was added to an equal volume of sample, standard or blank, and incubated at 25°C for 2 hours. MPs were isolated using a magnet, and unbound material was removed by washing with assay diluent using a HydroFlex plate washer (Tecan Group AG, Männedorf, Switzerland). Fluorescently-labeled detection antibody (20 μl, 100 pg/ml) was added to each well. MPs bearing the antibody–oligomer sandwich were then incubated with agitation using a Jitterbug shaker (Boekel, Feasterville, PA, USA) for 1 hour at 25°C. Unbound detection reagent was removed by washing (four times) with assay buffer, and MPs were transferred to a new plate. The assay buffer was removed by aspiration, and fluorescently-labeled detection antibody released by shaking in elution buffer (10 μl/well) for 5 minutes at 25°C. Eluates were then transferred to the wells of a 384-well plate containing neutralization buffer (10 μl/well). The sample was then drawn into an Erenna instrument and passed through a 100 μm diameter capillary flow cell through which a laser is directed. When fluorescently-labeled antibodies pass through the interrogation space, they emit light that is measured using a confocal microscope lens and a photon detector. The output from the detector is a train of pulses, with each pulse representing one photon that was detected. In this way, three signal outputs can be obtained: detected events (low end signal), event photons (low and medium end signal), and total photons (high end signal). For our experiments, we focused on detected events, generating standard curves from detected event values using a four-parameter curve fit. LOD and LLoQ were calculated as described for the MSD-based assay.

### Measurements of tau, phosphorylated tau and Aβ1–42 in CSF

Samples in Cohort 1 were analyzed for Aβ42, t-tau and P-tau181 at the Clinical Neurochemistry Laboratory, Sahlgrenska University Hospital, Mölndal, Sweden, by board-certified laboratory technicians using commercially available INNOTEST ELISA kits (Fujirebio Europe, Ghent, Belgium). All analytical procedures were performed according to protocols accredited by the Swedish Board for Accreditation and Conformity Assessment.

CSF samples in Cohort 2 and Cohort 3 were analyzed for Aβ42, t-tau and P-tau181 with an Inno-Bia Alzbio3 kit (Fujirebio Europe) following the manufacturer’s protocol, and were read on a FlexMap3D platform (Luminex Corporation, Austin, TX, USA).

### Human brain homogenate preparation

Homogenates of human brains were prepared as described elsewhere [17]. Frozen cortices were provided by Dr M Frosch (MGH and MADRC Neuropathology Core, Harvard, MA, USA) under institutional review board-approved protocols. Frozen samples of temporal or frontal cortex (1 g) were allowed to thaw on ice, chopped into small pieces with a razor blade, and then homogenized with 25 strokes of a Dounce homogenizer (Fisher, Ottawa, ON, Canada) in 4 ml ice-cold 20 mM Tris–HCl, pH 7.4, containing 150 mM NaCl (Tris-buffered saline (TBS)) and protease inhibitors. Water-soluble Aβ was separated from membrane-bound and plaque Aβ by centrifugation at 175,000 × *g* and 4°C in a TLA 100 rotor (Beckman Coulter, Fullerton, CA, USA) for 30 minutes, and the supernatant (referred to as TBS extract) aliquoted and stored at −80°C. The ethical body approving this study was the Partners Institutional Review Board of the Partners Human Research Committee.

### Cerebrospinal fluid sample collection and processing

Samples were collected from the L3/L4 interspace [18] and transferred into nonabsorbing (polypropylene) tubes. For Cohorts 1 and 2, CSF was mixed by gently inverting three or four times and then transferred to polypropylene storage tubes and stored at −80°C. For Cohort 3, immediately following collection, CSF samples were mixed by gently inverting three or four times and then centrifuged at 400 × *g* for 10 minutes. The crystal-clear supernatant was removed to a polypropylene tube and centrifuged at 2,000 × *g* at 4°C for 10 minutes, and aliquots of the supernatant were transferred to polypropylene storage tubes and stored at −80°C.

## Results

### Developing an Aβ oligomer-selective immunoassay with improved sensitivity

In an attempt to achieve an assay that can sensitively detect soluble oAβ in human biological fluids, we systematically evaluated several mAbs and two detection platforms. We initially searched for a preferred antibody pair using the MSD Model Sector Imager 2400A platform (MesoScale Discovery). We compared four antibody combinations, using unlabeled antibodies for capture and biotinylated (B) antibodies for detection: 1C22/3D6B, 1C22/82E1B, 3B3/3D6B (3B3 is the murine precursor of the humanized 19.3 mAb used by Savage and colleagues [8]), and 3B3/82E1B (Table 1). We tested the ability of each combination to detect three different synthetic Aβ preparations: ADDLs, a complex mixture of partially aggregated but still soluble oligomers of Aβ1-42 and unaggregated Aβ monomer (Additional file 1) [19]; (S26C)_2_, a covalently cross-linked dimer formed by oxidation of Aβ1-40Ser26Cys that upon freeze–thawing contains both authentic dimers and higher aggregates of dimers (Additional file 2) [20]; and DiY, an Aβ1-40 dimer formed by covalent phenolic coupling of two tyrosine-10 residues that has a low propensity for further aggregation [14] (Additional file 2). Since the DiY standard does not further assemble under the conditions used, the absolute molar concentration of this dimer standard is known. However, since (S26C)_2_ can further aggregate and, like ADDLs, contains a heterogeneous mixture of assemblies (Additional files 1 and 2), the molar concentrations of these standards are expressed in terms of dimer and monomer, respectively.

**Table 1:** **Anti-human amyloid-beta IgG monoclonal antibodies**

**Antibody**	**Immunogen**	**Epitope**
1C22	Aggregated (Aβ(1–40)S26C)_2_	Aβ oligomer mix
3B3	Aβ1–42 ADDL preparation	Aβ oligomer mix
3D6	Aβ(1–5) synthetic peptide	Requires Aβ(1)
82E1	Aβ(1–16) synthetic peptide	Requires Aβ(1)

Aβ, amyloid beta; (Aβ(1–40)S26C)_2_, cystine cross-linked Aβ dimer; ADDL, amyloid-beta-derived diffusible ligand.

LLoQ, defined as the lowest back-interpolated concentration of standard that provided a signal two-fold greater than background and had a coefficient of variation <20%, was determined for each mAb pair with each synthetic Aβ preparation.

**Figure 1:**
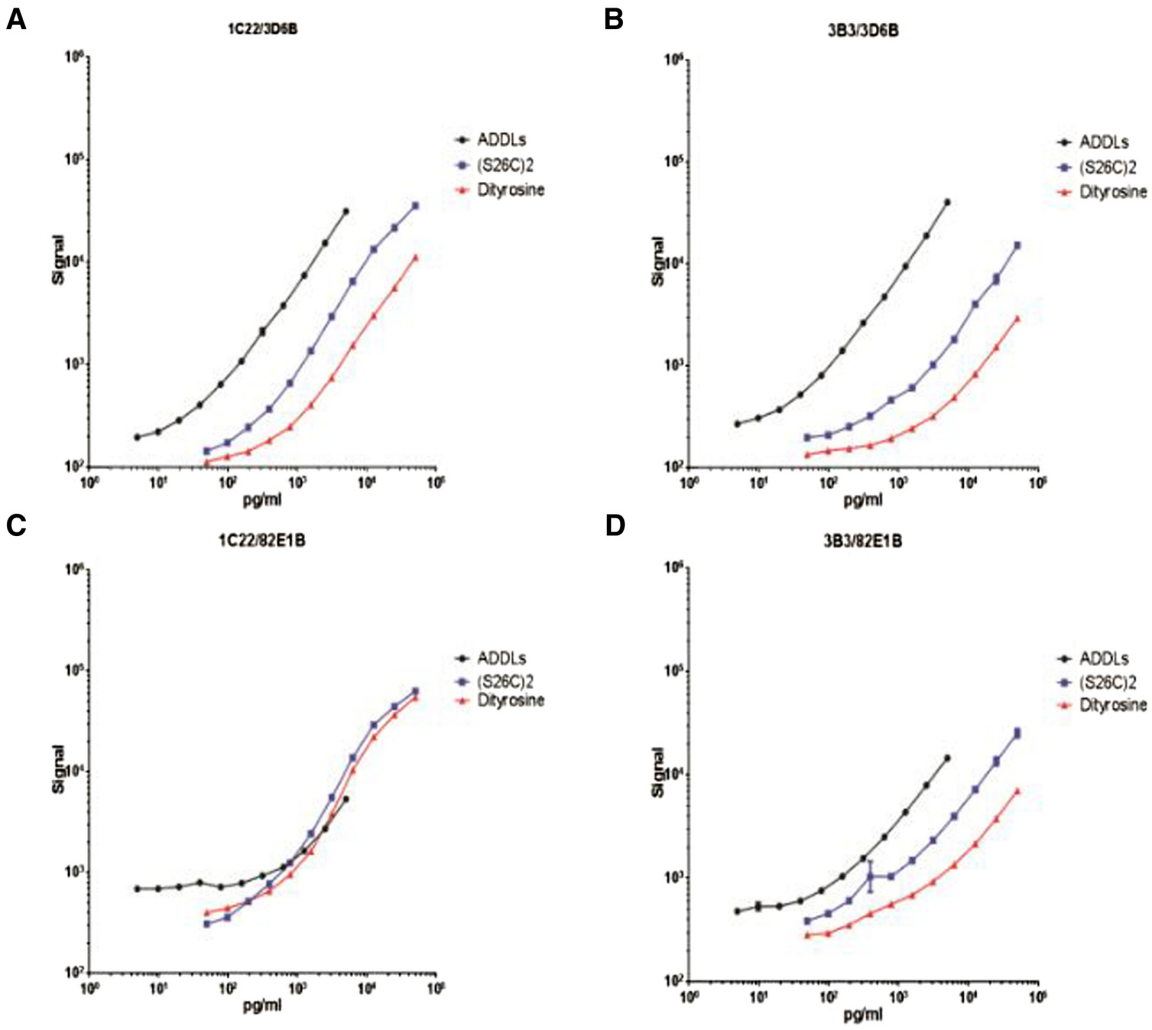
**Comparison of antibody pairs in amyloid-beta oligomer enzyme-linked immunosorbent assay on the MSD platform.** Four antibody pairs – 1C22/3D6B **(A)**, 3B3/3D6B **(B)**, 1C22/82E1B **(C)** and 3B3/82E1B **(D)** – were tested on the MSD platform (MSD Multi-Array; Meso Scale Discovery, Gaithersburg, MD, USA) for their ability to detect either amyloid-beta-derived diffusible ligands (ADDLs; black circles), the pro-aggregating (S26C)_2_ dimer (blue squares) or the aggregation-resistant dityrosine dimer (red triangles). Note that signal units on the *y* axis and concentrations of the amyloid-beta oligomers (pg/ml) on the *x* axis are on a log scale.

Using the MSD platform, the 1C22/3D6B assay allowed the most sensitive detection of ADDLs, with LLoQ = 18.7 pg/ml. The 1C22/82E1B demonstrated the lower sensitivity for detecting ADDLs (LLoQ = 598.6 pg/ml), whereas the 3B3/3D6B (LLoQ = 37.4 pg/ml) and 3B3/82E1B (LLoQ = 74.7 pg/ml) assays gave intermediate sensitivities (Figure 1A,B,C,D). Three out of the four antibody pairs detected (S26C)_2_ and DiY much less sensitively than ADDLs (Figure 1A,B,D), and in all cases DiY was the least well detected species (Figure 1A,B,C,D and Table 2).

**Table 2:** **Comparison of four antibody pairs for sensitivity to detect three different synthetic amyloid-beta oligomer preparations on the MSD platform**

		**1C22/3D6B**	**1C22/82E1B**	**3B3/3D6B**	**3B3/82E1B**
ADDLs	LoD	4.0	258.6	3.2	51.6
	LLoQ	18.7	598.6	37.4	74.7
(S26C)_2_	LoD	55.6	48.7	84.0	26.8
	LLoQ	187.0	390.0	390.6	390.6
DiY	LoD	154.0	92.7	369.0	44.8
	LLoQ	601.0	390.6	1562.5	781.3

ADDL, amyloid-beta-derived diffusible ligand; DiY, dityrosine; LLoQ, lower limit of reliable quantification; LOD, limit of detection; MSD platform. MSD Multi-Array (Meso Scale Discovery, Gaithersburg, MD, USA).

Overall the 1C22/3D6B mAb pair most sensitively detected the three forms of synthetic oAβ tested and thus was selected for further study.

### Comparison of sensitivities between the MSD and Erenna platforms for different synthetic Aβ oligomers

To improve the sensitivity of the oligomer-specific enzyme-linked immunosorbent assay (o-ELISA) for the low levels of oAβ likely to be present in human CSF [3,9], we employed the highly sensitive Erenna detection platform, as was used by Savage and colleagues [8,16]. We again examined detection of ADDLs, (S26C)_2_ and DiY using 1C22 for capture and fluorescently-labeled 3D6 for detection. As we saw in our preliminary MSD experiments (Figure 1), the most highly aggregated synthetic species, ADDL, was most sensitively detected in both the MSD and Singulex platforms (Table 3). On the MSD platform, concentrations of our ADDL standards ranged from 4,789 to 4.67 pg/ml, and the LLoQ (defined as above) was 18.7 pg/ml. On the Erenna platform, ADDL standards ranged from 10 to 0.0094 pg/ml, and the LLoQ was 0.15 pg/ml. The Erenna platform thus allowed detection of ADDLs at a concentration 125 times lower than the MSD platform. The (S26C)_2_ standard contained smaller and fewer aggregates than the ADDL standard (compare Additional file 1 and Additional file 2) and was less well detected than ADDLs, with LLoQs of 40.6 ± 9.3 pg/ml on the Erenna platform and 187 ± 54 pg/ml on the MSD platform. The DiY covalent dimer, which under the conditions used contains only dimers (Additional file 2), was least well detected with the Erenna and MSD platforms, yielding LLoQs of 375 ± 84.6 pg/ml on the Erenna platform and 601 ± 107 pg/ml on the MSD platform.

**Table 3:** **Comparison of sensitivities (LLoQ) of the 1C22/3D6B amyloid-beta oligomer assay using the MSD versus Erenna platforms**

**ELISA platform**	**ADDLs (pg/ml)**	**(S26C)**_**2**_ **(pg/ml)**	**Dityrosine (pg/ml)**
Erenna	0.15	40.6 ± 9.3	375 ± 84.6
MSD	18.7	187 ± 54	601 ± 107

LLoQ is the lowest back-interpolated standard that provides a signal two-fold greater than background with a coefficient of variance <20%. ADDL, amyloid-beta-derived diffusible ligand; ELISA, enzyme-linked immunosorbent assay; LLoQ, lower limit of reliable quantification. Erenna platform (Singulex, Alameda, CA, USA) and MSD Multi-Array (Meso Scale Discovery, Gaithersburg, MD, USA).

Based on these sensitivity results, we chose to employ ADDLs as our Aβ standard and use the Erenna platform in all subsequent experiments.

### Selectivity of the oligomer-specific ELISAs versus Aβ monomer

**Figure 2:**
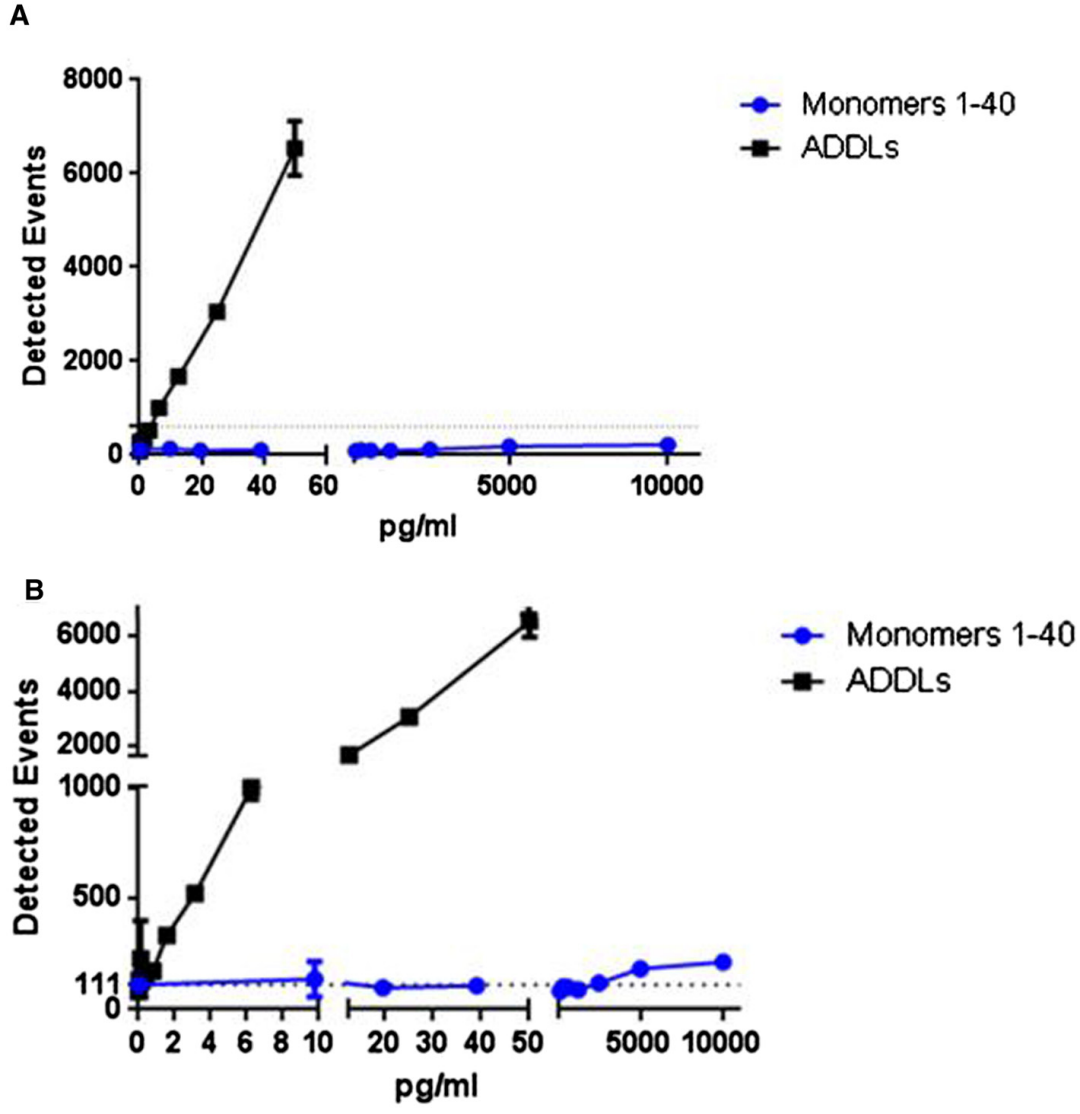
**1C22/3D6 oligomer-specific ELISA on the Erenna platform specifically recognizes amyloid-beta oligomers, not monomers. (A)** Amyloid-beta monomers show virtually no reactivity in the 1C22/3D6 oligomeric-specific enzyme-linked immunosorbent assay: 26,000-fold lower signal compared with oligomers. **(B)** Lowest end of the detection range in (A) expanded to visualize the minimal detection of monomers at very high concentrations. Dashed line at 111 on the *y* axis, signal of blank. ADDL, amyloid-beta-derived diffusible ligand. Erenna platform (Singulex, Alameda, CA, USA).

Cross-reactivity of the Aβ o-ELISA assay with Aβ monomers was assessed using carefully prepared monomer solutions of synthetic Aβ1–40 that were devoid of any detectable aggregates (Figure S2A,C,E in Additional file 2). At monomer concentrations that ranged from 10,000 pg/ml down to 9.77 pg/ml there was virtually no signal detectable, whereas even at concentrations below 1 pg/ml synthetic ADDLs were readily detected (Figure 2). Percent cross-reactivity was calculated by dividing the interpolated values by the expected values based on an oligomer standard curve at comparable protein concentrations. The calculated percent cross-reactivity for monomers was 0.003% at 5,000 pg/ml Aβ40 monomer and 0.005% at 10,000 pg/ml Aβ40 monomer. Thus, even at these very high monomer concentrations, the 1C22/3D6 Erenna assay demonstrated ~26,000-fold higher selectivity for ADDLs versus monomer. Moreover, given that the concentration of oligomers in our ADDL standard is expressed in terms of monomer content, but that oligomers by definition are composed of two or more monomers, it is evident that the actual molar concentration of oligomers in both our standards and samples is significantly lower than the values based on monomer content. These results indicate that the 1C22/3D6 Erenna o-ELISA is very highly specific for oligomers and does not significantly detect monomeric Aβ.

We did not perform selectivity analyses of the 1C22/3D6 ELISA on Aβ42 monomer using the Erenna platform, but did perform such analyses using the MSD platform, and these results (data not shown) indicate much greater selectivity of the ELISA for oligomers over monomers, although as expected Aβ42 (but not Aβ40) can spontaneously form oligomers at very high Aβ42 concentrations, consistent with the fact that this longer peptide is well known to be much more prone to oligomerization. However, our CSF samples do not contain these very high concentrations of Aβ42, which exists at 1/10 the concentration of Aβ40 in normal CSF and is even lower than that in AD and MCI CSF.

### Characterization of the oligomeric Aβ species detected by oligomer-specific ELISA

**Figure 3:**
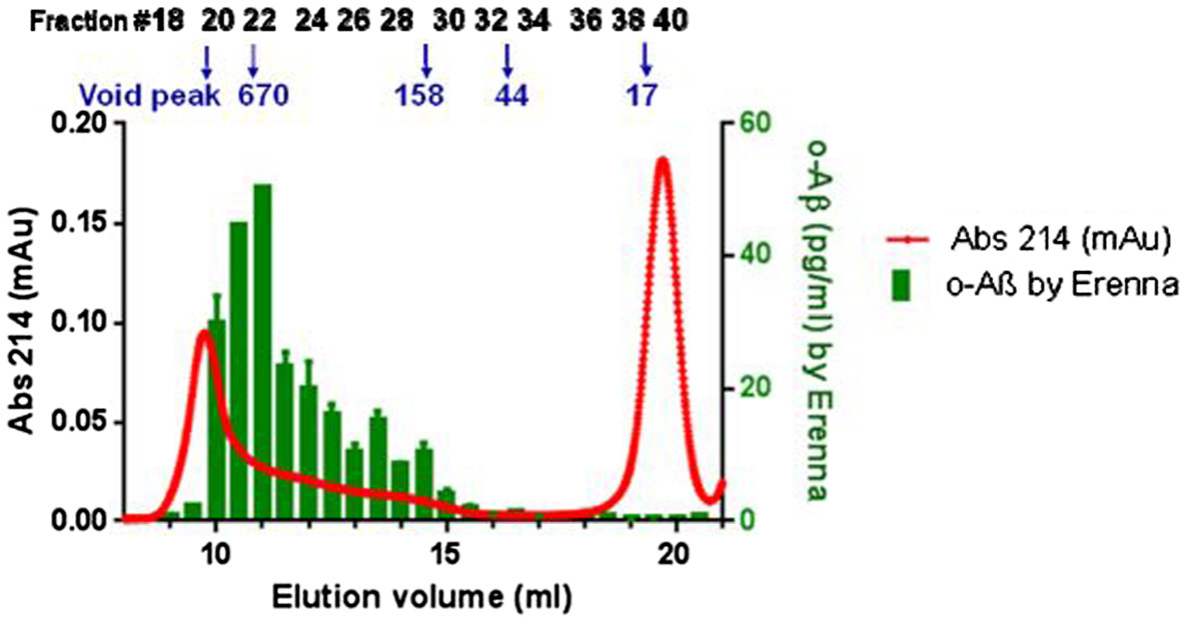
**Analysis of size exclusion chromatography-fractioned ADDLs reveals that the oligomer-specific ELISA detects a range of different sized amyloid-beta assemblies but not monomers.** The amyloid-beta-derived diffusible ligand preparation was chromatographed on a Superdex 200 10/30 HR column (GE Healthcare Biosciences, Pittsburgh, PA, USA) by eluting in sodium phosphate buffer at 0.5 ml/minute. Absorbance at 214 nm was recorded, and 0.5 ml fractions were collected and analyzed by the 1C22/3D6 Erenna oligomer-specific enzyme-linked immunosorbent assay (o-ELISA). All fractions were diluted 5 × 10^5^-fold prior to o-ELISA. Elution positions of Blue Dextran and globular standards are indicated by arrows and molecular weights (kDa). oAβ, amyloid-beta oligomers. Erenna platform (Singulex, Alameda, CA, USA).

The differential detection of ADDLs versus (S26C)_2_ and DiY (Figure 1) suggested that our assay preferentially detects large versus small oAβ. Thus, to clarify what range of sizes of oAβ can be detected by our o-ELISA, ADDLs were fractionated by SEC on a Superdex 200 column (molecular weight range: 3,000 to 600,000 Da) and the fractions quantified using the 1C22/fluorescently-labeled 3D6 Erenna immunoassay. The UV absorption chromatogram produced by the ADDLs demonstrated the presence of a range of different-sized assemblies (Figure 3, red line). These included a broad trailing peak that began close to the void volume of the column and eluted over a further ~7 ml (spanning fractions 18 to 32), and a monomer peak centered at ~19.5 ml (fractions 37 to 42). Immediately following collection, the fractions were diluted 2 × 10^5^-fold and assayed by the o-ELISA (Figure 3, green bars). The assay detected soluble oligomers in fractions that spanned a broad range of oligomer sizes ranging from >600 kDa down to ~40 kDa (relative to globular protein standards), but it was insensitive to the Aβ monomer fractions. The similar patterns between the UV and o-ELISA chromatograms (Figure 3) indicates that the o-ELISA recognizes a broad size range of oAβ of masses greater than ~40 kDa. In addition, the o-ELISA can detect true dimers of DiY Aβ (see Figure 1A). The lack of reaction with SEC-isolated monomers further confirms the strong selectivity of the o-ELISA for oligomers.

### Application to natural human oligomers: dilution linearity of the Aβ oligomer signal in AD-TBS brain extracts

**Figure 4:**
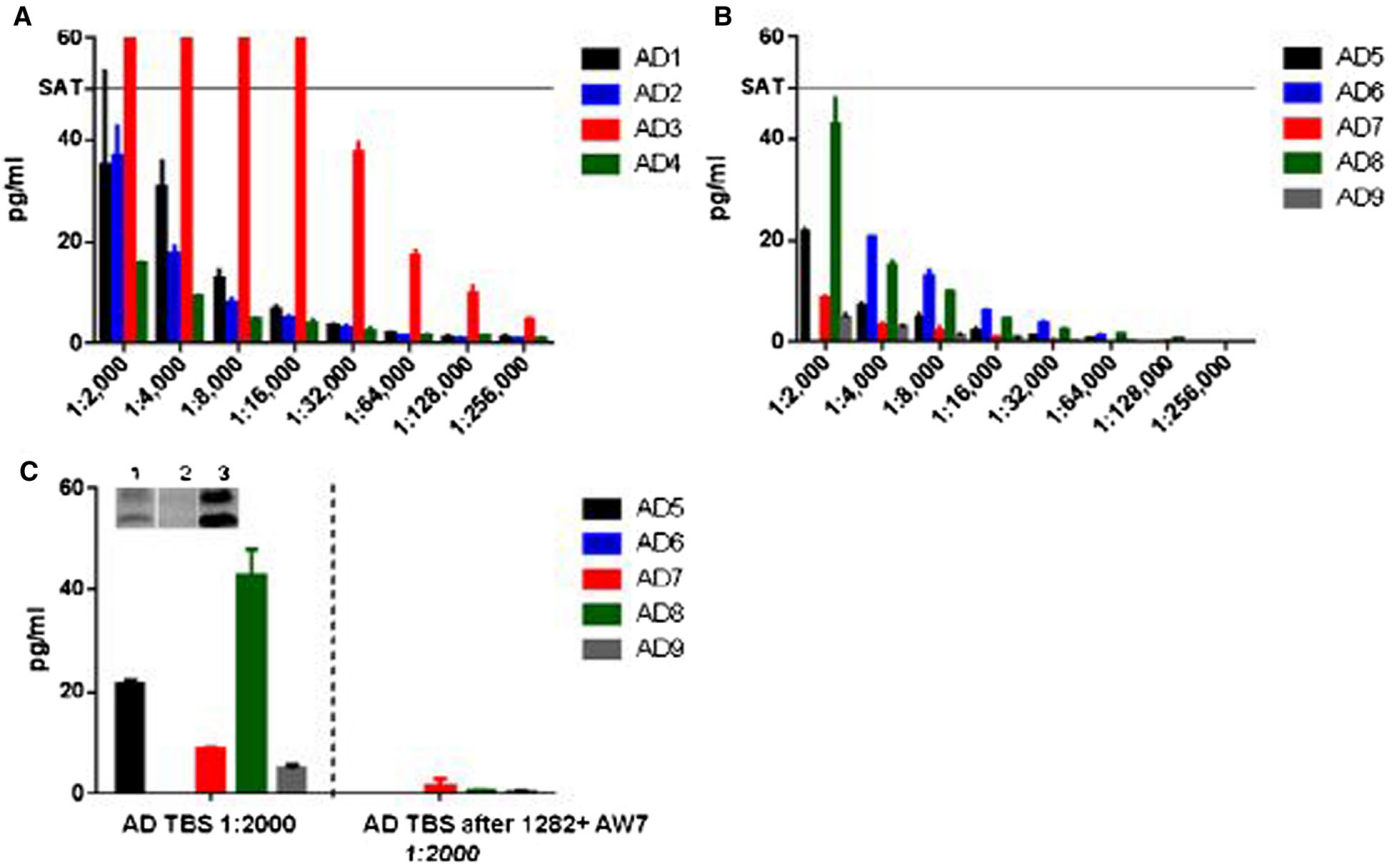
**Oligomer-specific ELISA specifically detects human amyloid-beta species in AD-TBS brain extracts.** Soluble extracts of Alzheimer’s disease (AD) brains 1 to 4 **(A)** and brains 5 to 9 **(B)** were serially diluted and assayed with the Erenna 1C22/3D6 oligomer-specific enzyme-linked immunosorbent assay (o-ELISA), revealing highly linear concentration curves in the extracts of all brains. SAT, o-ELISA value of the highest loaded standard (50 pg/ml). **(C)** After pre-clearing with plain beads, AD-TBS extracts of brains 5 to 9 were each subjected to sequential IPs with amyloid-beta antisera 1282 and AW7, and the resultant supernatants assayed by o-ELISA. Western blotting (inset: monoclonal antibodies 6E10 + 2G3 + 21 F12) of a representative immunodepletion experiment shows: lane 1, starting AD extract; lane 2, its supernatant after 1282 + AW7 immunodepletion; and lane 3, IP’ed 1282 + AW7 pellet. TBS, Tris-buffered saline (20 mM Tris–HCl, pH 7.4, containing 150 mM NaCl). Erenna platform (Singulex, Alameda, CA, USA).

We next sought to establish the usefulness of the o-ELISA for quantifying natural oligomers of human Aβ found in aqueous extracts of AD brain tissue (Figure 4). To this end, we analyzed extracts from nine different brains with clinically and neuropathologically confirmed AD, diluting each extract serially from 1:2,000 to 1:256,000. The signal detected in each extract decreased linearly and appropriately upon dilution (Figure 4A,B and Table 4). Moreover, when extracts from five of the brains were examined before and after immunodepletion with the combined pan-Aβ antisera R1282 and AW7, little or no o-ELISA signal remained (Figure 4C). The effectiveness of the immunodepletion was confirmed by western blotting (Figure 4C, inset). In our previous work [3], Aβ1–X monomer levels were reported at 165 ± 48 pg/g wet tissue in the nine controls whose ages matched those of our AD patients (that is, >55 years old) versus 1,995 ± 1,217 pg/g in the 13 AD patients. The monomer is thus very unlikely to be detectable by the o-ELISA, even in our lowest dilution of 1:2,000, so we can exclude interference from Aβ monomer levels in the brain extracts. Taken together, these results indicate that the o-ELISA can detect natural oligomers over a wide range of pathophysiologically relevant concentrations in AD cortical samples and that the signals are derived specifically from Aβ.

**Table 4:** **Raw data for Figure** 4 **: mean amyloid-beta oligomer values in serial dilutions of AD-TBS extracts from nine brains using the 1C22/3D6B oligomer-specific ELISA**

**Dilutions**	**AD1 (pg/ml)**	**AD2 (pg/ml)**	**AD3 (pg/ml)**	**AD4 (pg/ml)**	**AD5 (pg/ml)**	**AD6 (pg/ml)**	**AD7 (pg/ml)**	**AD8 (pg/ml)**	**AD9 (pg/ml)**
1:2,000	35.0 ± 18.7	36.8 ± 6	SAT	15.5 ± 0.3	21.5 ± 0.8	SAT	8.7 ± 0.4	42.9 ± 5.0	4.7 ± 0.8
1:4,000	31.0 ± 4.8	17.7 ± 1.4	SAT	9.2 ± 0.4	7.1 ± 0.6	20.8 ± 0.05	3.2 ± 0.5	15.0 ± 0.8	2.9 ± 0.3
1:8,000	12.9 ± 1.6	8.0 ± 0.9	SAT	4.9 ± 0.2	4.9 ± 0.6	13.0 ± 1.16	2.2 ± 0.8	9.9 ± 0.3	1.3 ± 0.2
1:16,000	7.0 ± 0.5	5.0 ± 0.3	SAT	4.0 ± 0.5	2.2 ± 0.4	6.1 ± 0.31	0.9 ± 0.1	4.6 ± 0.1	0.8 ± 0.2
1:32,000	3.5 ± 0.3	2.9 ± 0.6	37.5 ± 2.2	2.5 ± 0.5	1.1 ± 0.1	3.6 ± 0.42	0.4 ± 0.1	2.5 ± 0.1	0.3 ± 0
1:64,000	2.0 ± 0.2	1.4 ± 0.1	17.1 ± 1.2	1.6 ± 0.1	0.6 ± 0.1	1.3 ± 0.1	ND	1.4 ± 0.2	0.4 ± 0
1:128,000	1.2 ± 0.3	0.9 ± 0.2	10.0 ± 1.4	1.4 ± 0.1	ND	ND	0.4	0.6 ± 0.1	ND
1:256,000	1.2 ± 0.4	0.9 ± 0.1	4.6 ± 0.3	1.0 ± 0.2	ND	ND	ND	ND	ND
AD-TBS (1:2,000) after 1282 + AW7 immunodepletion	N/A	N/A	N/A	N/A	ND	ND	1.4 ± 1.5	0.6 ± 0.1	0.4 ± 0.1

AD, Alzheimer’s disease; ELISA, enzyme-linked immunosorbent assay; SAT, amyloid-beta oligomer values are higher than the highest loaded standard (50 pg/ml); ND, amyloid-beta oligomer values are too low to be detectable; TBS, Tris-buffered saline (20 mM Tris–HCl, pH 7.4, containing 150 mM NaCl).

### Quantifiable levels of Aβ oligomers are present in most human CSF samples

**Figure 5:**
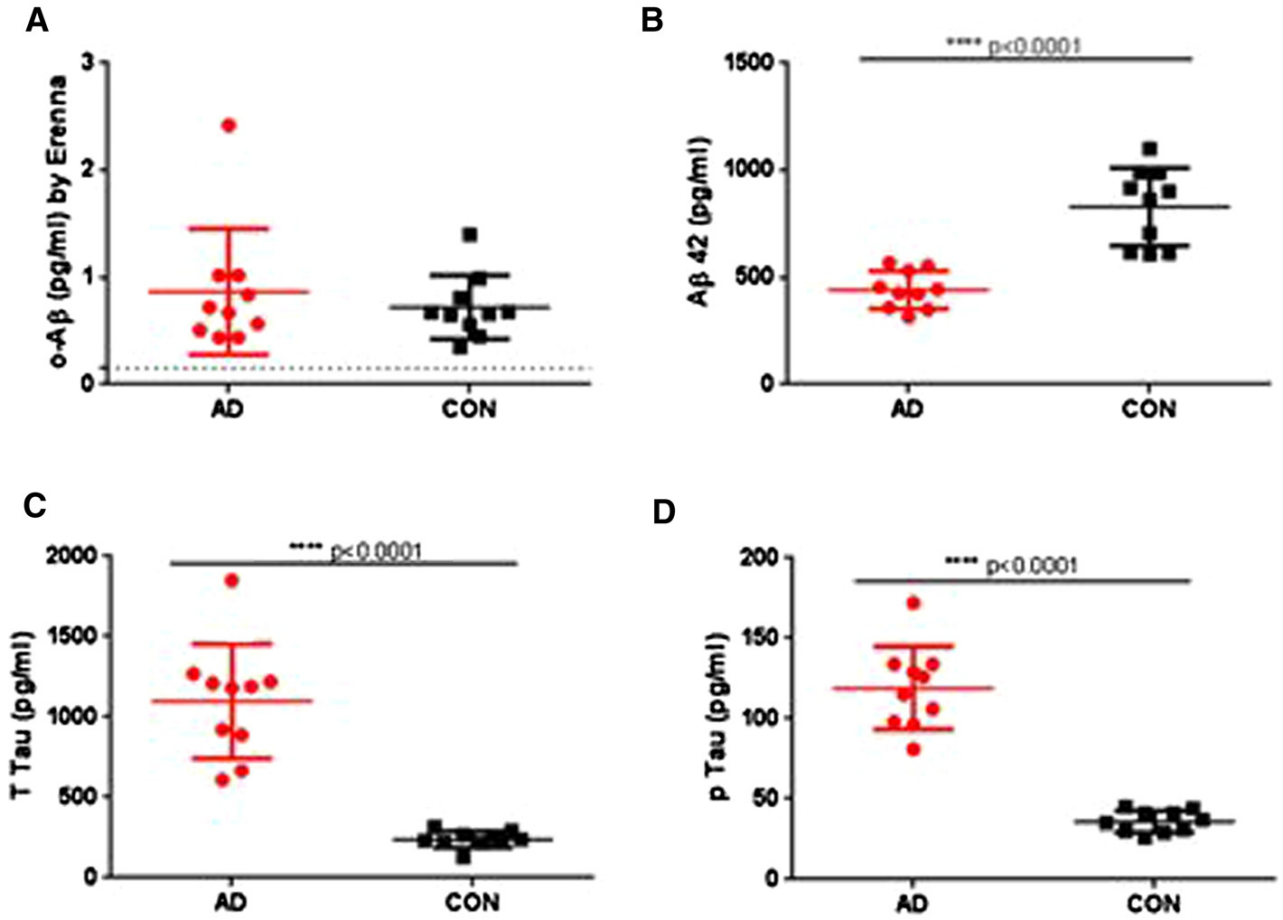
**Cerebrospinal fluid levels of Aβ42, t-tau and p-tau, but not amyloid-beta oligomers, distinguish Alzheimer’s disease subjects from age-matched controls in Cohort 1.** Cerebrospinal fluid (CSF) from 10 Alzheimer’s disease (AD) and 10 aged control (CON) subjects were analyzed for amyloid-beta oligomers (oAβ) **(A)**, Aβ42 **(B)**, total tau **(C)** and phosphorylated tau **(D)**. Aβ42 quantified with monoclonal antibodies (mAbs) 21 F12 for capture and 3D6 for detection (unpaired, two-tailed *t* test with Welch’s correction, *P* <0.0001). Total tau quantified with mAbs AT120 for capture and HT7 + BT2 for detection (unpaired, two-tailed *t* test with Welch’s correction, *P* <0.0001). Tau phosphorylated at Thr181 quantified with of HT7 for capture and AT120 for detection (unpaired, two-tailed *t* test with Welch’s correction, *P* <0.0001). Horizontal bars, medians and interquartile ranges. Dashed line crosses *y* axis at 0.15 pg/ml, indicating the lower limit of reliable quantification. Erenna platform (Singulex, Alameda, CA, USA).

Having established that the 1C22/fluorescently-labeled 3D6 Erenna o-ELISA is highly specific and can detect a range of different-sized Aβ assemblies, including natural oligomers present in all AD brain extracts tested, we asked whether this assay could detect oligomers in human CSF. Our initial analysis used CSF from 10 AD subjects and 10 healthy controls. These samples were selected based on Aβ42, t-tau and p-tau values so that the levels for all three markers in the AD group exceeded cutoff points proposed as being diagnostic for AD [21], whereas the values for all three markers in the non-AD group did not meet the cutoff points for AD (Figure 5B,C,D). These samples were collected at Sahlgrenska University Hospital (Mölndal, Sweden) and are referred to as Cohort 1 (Table 5 and Figure 5A). In this cohort, oAβ levels ranged from 0.35 to 2.42 pg/ml, with all values greater than the LLoQ of 0.15 pg/ml. To confirm that the immunoassay signals derived specifically from oAβ, two representative CSF samples were immunoprecipitated using a combination of R1282 and AW7 and the supernatants subjected to o-ELISA alongside the unmanipulated starting CSF. After this immunodepletion, virtually no oligomeric Aβ signal was detected (Table 6), a result identical to that of the brain extracts (Figure 4C), thus indicating that the signal detected in the human CSF samples is attributable to authentic oAβ.

**Table 5:** **Demographic data and biomarker concentrations for all cerebrospinal fluid samples**

	**Diagnosis**	***n***	**Sex (male/female)**	**Age (years)**	**MMSE score**	**Aβ1–42 (pg/ml)**	**Total tau (pg/ml)**	**Phosphorylated tau (pg/ml)**	**oAβ (pg/ml)**
Cohort 1	AD	10	3/7	72	N/A	442.6 ± 27.4	1101 ± 112.5	119.1 ± 8.2	0.9 ± 0.2
				(54 to 84)		(316, 568)	(609, 1,850)	(81,172)	(0.4, 2.4)
	Normal	10	5/5	72	N/A	829.8 ± 57.3	239.5 ± 15.8	35.9 ± 2.1	0.7 ± 0.1
				(62 to 81)		(607, 1,100)	(129, 315)	(26, 45)	(0.4, 1.4)
Cohort 2	AD	10	6/4	72	18	184.4 ± 11.2	154.9 ± 22.5	34.4 ± 3.2	0.2 ± 0.0
				(50 to 86)	(11 to 20)	(147, 236)	(70, 247)	(22, 47)	(0.1, 0.3)
	MCI	10	6/4	81	25	250.0 ± 25.6	142.3 ± 15.6	31.3 ± 4.3	0.3 ± 0.1
				(76 to 89)	(23 to 28)	(165, 383)	(80, 207)	(17, 59)	(0.1, 0.8)
	Normal	10	4/6	79	28	251.1 ± 34.1	58.8 ± 6.4	16.7 ± 1.8	0.2 ± 0.0
				(75 to 82)	(27 to 29)	(126, 399)	(36, 84)	(10, 25)	(0, 0.4)
Cohort 3	MCI	23	16/7	75	28	386.6 ± 30.0	152.4 ± 29.4	46.1 ± 5.1	0.9 ± 0.1
				(60 to 85)	(25 to 30)	(201, 599)	(39, 530)	(20, 90)	(0.3, 2.7)
	Normal	17	7/10	70	29	475.4 ± 23.1	95.5 ± 20.0	30.7 ± 5.3	0.6 ± 0.1
				(55 to 86)	(27 to 30)	(352, 590)	(26, 285)	(11, 77)	(0.3, 1.3)

Data presented as mean ± standard error of the mean (range). AD, Alzheimer’s disease; MCI, mild cognitive impairment; MMSE, Mini-Mental State Examination; oAβ, amyloid-beta oligomers.

**Table 6:** **Amyloid-beta oligomer levels in cerebrospinal fluid samples before versus after immunodepletion with two amyloid-beta antisera**

**CSF sample**	**oAβ (pg/ml)**
CSF 1	1.18 ± 0.21
CSF 1 after 1282	0.22 ± 0.25
CSF 1 after 1282 and AW7	ND
CSF 2	3.16 ± 0.31
CSF 2 after AW7	ND
CSF 2 after AW7 and 1282	0.05 ± 0.003

CSF, cerebrospinal fluid; ND, too low to detect; oAβ, amyloid-beta oligomers.

The oAβ values in the AD group of Cohort 1 completely overlapped with those in the control group (Figure 5A). Results from these clinically and biomarker well-characterized samples demonstrate that the current o-ELISA cannot discriminate between AD and controls.

**Figure 6:**
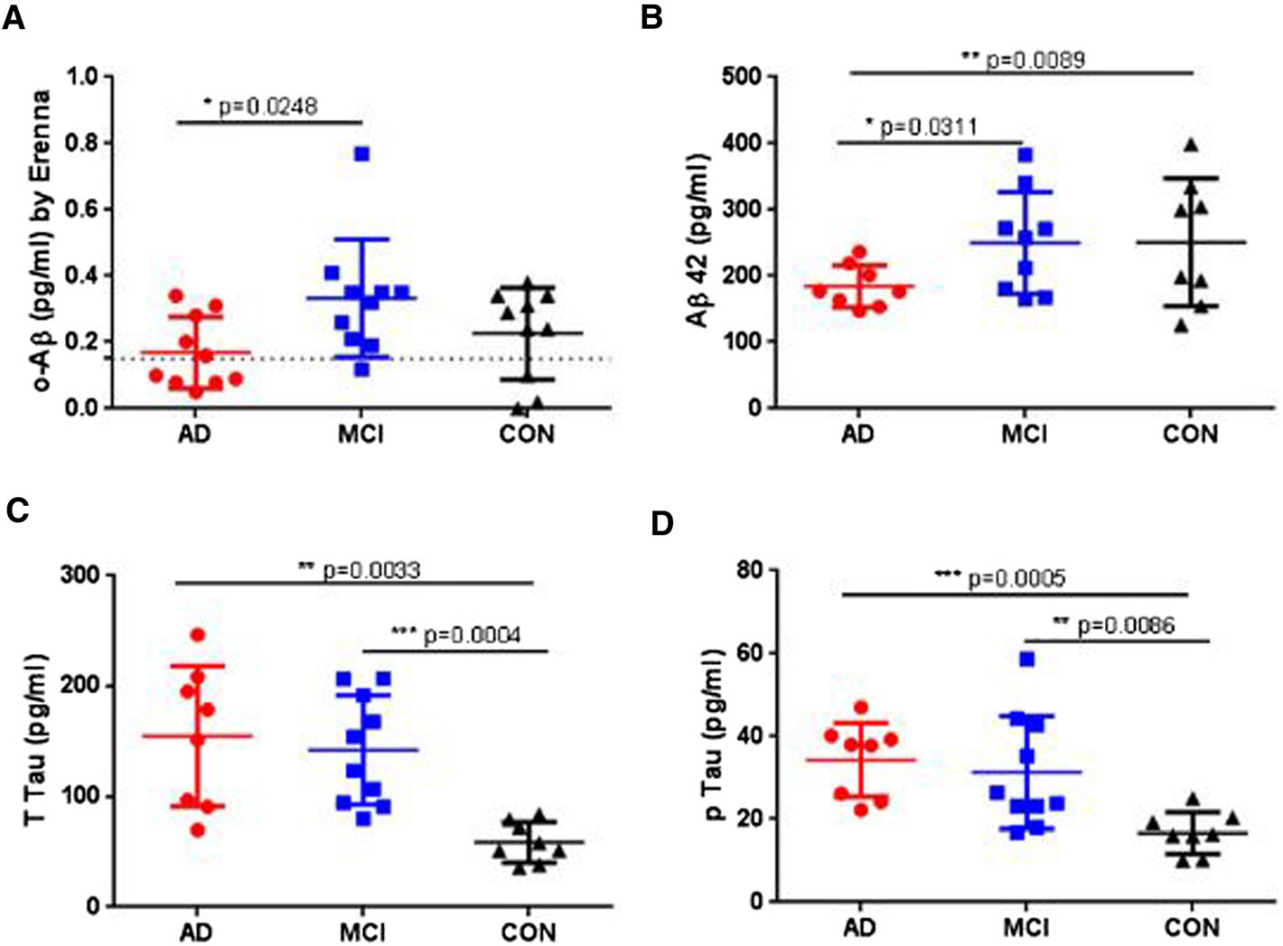
**Cerebrospinal fluid levels of amyloid-beta oligomers, Aβ42, t-tau and p-tau in Cohort 2.** Alzheimer’s disease subjects (AD, *n* = 10), mild cognitive impairment subjects (MCI, *n* = 10) and age-matched controls (CON, *n* = 10) were analyzed for amyloid-beta oligomers (oAβ) **(A)**, Aβ42 **(B)**, total tau (t-tau) **(C)** and phosphorylated tau (p-tau) **(D)**. Aβ42, t-tau and p-tau were measured with the Luminex platform (Luminex Corporation, Austin, TX, USA) and the AlzBio3 kit (Fujirebio Europe, Ghent, Belgium). This kit simultaneously measures Aβ1–42, t-tau and P-tau181 in 75 μl cerebrospinal fluid. Horizontal bars, medians and interquartile ranges. Dashed line crosses *y* axis at 0.15 pg/ml, indicating the lower limit of reliable quantification. Erenna platform (Singulex, Alameda, CA, USA).

To investigate whether oAβ levels measured by 1C22/fluorescently-labeled 3D6 Erenna o-ELISA can differentiate between MCI patients and healthy controls, we analyzed a second set of CSFs. These included samples from 10 patients with amnestic-type MCI, 10 patients with AD and 10 healthy controls, all from Mayo Clinic (Rochester, MN, USA) and designated Cohort 2 (Table 5). Quantifiable levels of oAβ were detected in most (21 out of 30) Cohort 2 samples, with values ranging from below the LLoQ (0.15 pg/ml) to 0.77 pg/ml (Figure 6A). The values obtained from all three groups showed significant overlap, but the MCI group had higher mean oAβ levels (0.33 ± 0.06 pg/ml) than the AD group (0.17 ± 0.03 pg/ml) (unpaired, two-tailed *t* test with Welch’s correction, *P* = 0.0248), and the MCI group also trended higher than the control group (0.23 ± 0.04 pg/ml) but without significant difference (Figure 6A). In keeping with the results obtained in Cohort 1, t-tau and p-tau each discriminated AD and MCI from control, and Aβ42 discriminated AD from both MCI and control but not MCI from control (Figure 6B,C,D). Thus, the accepted AD biomarkers again discriminate between AD and controls, whereas the current o-ELISA does not.

**Figure 7:**
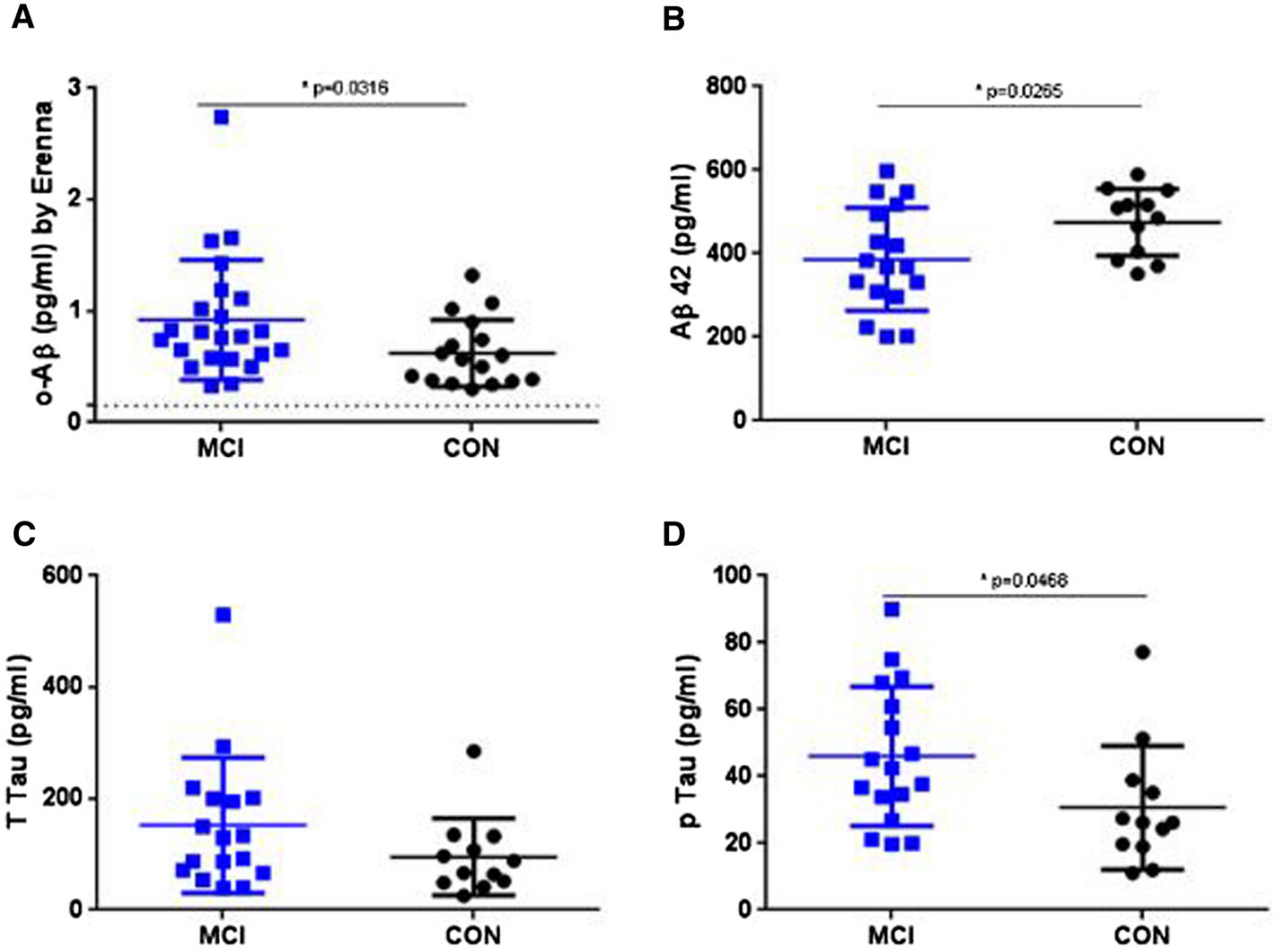
**Cerebrospinal fluid levels of amyloid-beta oligomers, Aβ42, t-tau and p-tau in Cohort 3.** Mild cognitive impairment subjects (MCI, *n* = 23) and age-matched controls (CON, *n* = 17) were analyzed. Significant differences are indicated. Labeling as in Figure 6. oAβ, amyloid-beta oligomers. Erenna platform (Singulex, Alameda, CA, USA).

Given that the MCI group in Cohort 2 tended to have higher oAβ levels than either the AD or control group, we analyzed a further 40 CSF samples: 23 from patients with MCI and 17 from healthy controls. These samples were obtained from the Harvard Biomarker Study collection and are designated Cohort 3 (Table 5). Again, quantifiable levels of oAβ were detected in all samples, with values ranging from 0.30 to 2.74 pg/ml and with considerable overlap between the two diagnostic groups (Figure 7A). Despite this overlap, mean levels of oAβ in the MCI group (0.92 ± 0.11 pg/ml) were significantly higher than those detected in the control group (0.62 ± 0.07 pg/ml) (*P* = 0.0316, unpaired, two-tailed *t* test with Welch’s correction). In this cohort, Aβ42 and p-tau assays again discriminated MCI from controls better than the o-ELISA. Interestingly, while p-tau levels were significantly different between the MCI patients and controls of Cohort 3, the difference was not as pronounced as those seen in Cohort 2, and t-tau levels were not significantly different between the MCI and control groups of Cohort 3.

**Figure 8:**
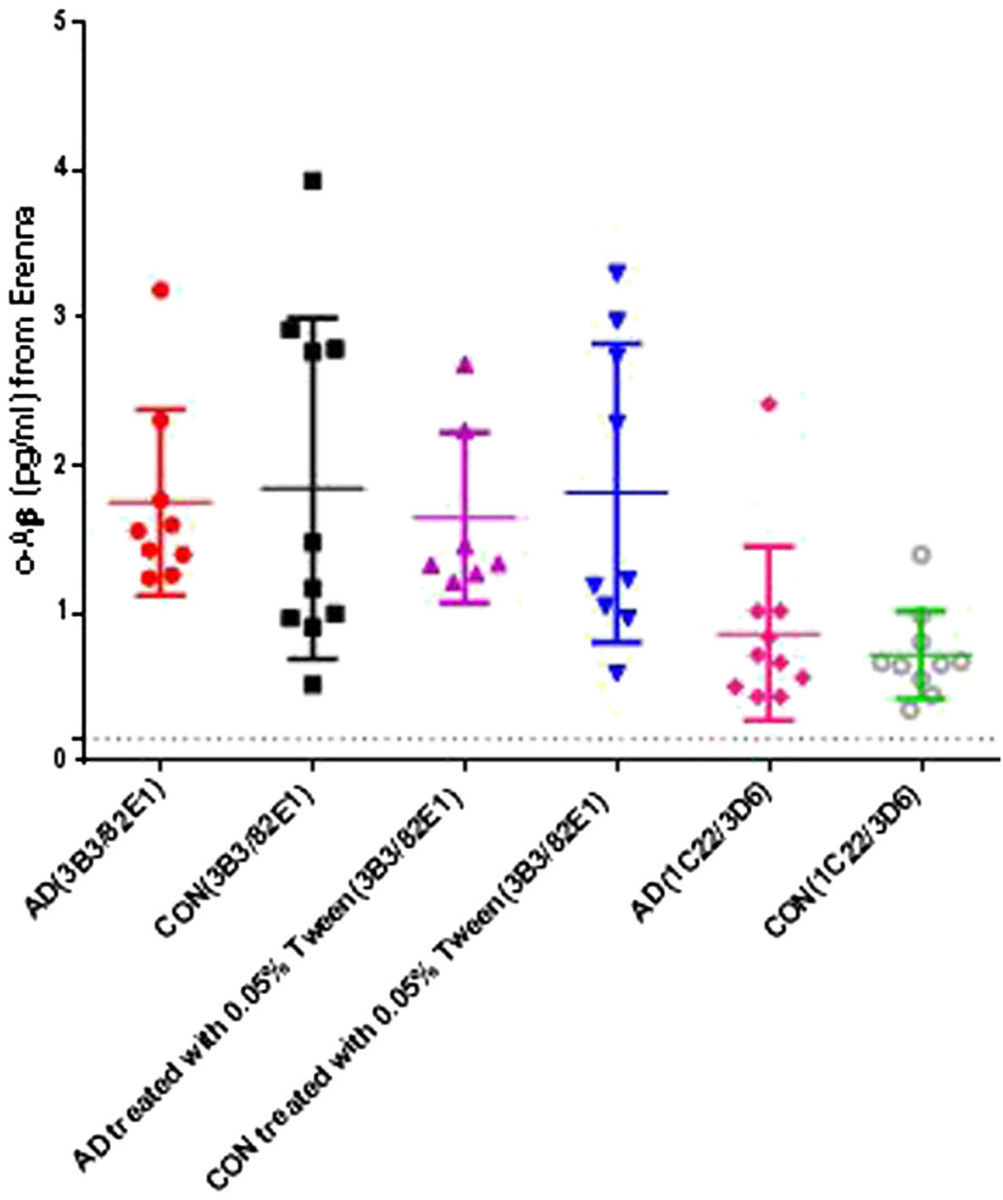
**Quantifying amyloid-beta oligomers in cerebrospinal fluid of Cohort 1 using two different monoclonal antibody pairs.** No significant differences in amyloid-beta oligomer (oAβ) levels between Alzheimer’s disease subjects (AD, *n* = 9) and controls (CON, *n* = 10) were obtained with either antibody pair or without versus with 0.05% Tween 20 in the samples. Labeling as in Figures 5, 6, and 7. Lower limit of reliable quantification for 1C22/3D6 and 3B3/82E1 was 0.15 pg/ml and 0.3 pg/ml, respectively; former indicated by a dashed line. Erenna platform (Singulex, Alameda, CA, USA).

In the two cohorts for which Mini-Mental State Examination (MMSE) scores were available (Cohorts 2 and 3), we evaluated whether there was any correlation between MMSE scores and oAβ levels. No significant correlations were found in any of the control, AD or MCI groups. MMSE scores were not available for Cohort 1. To directly compare our results with those of Savage and colleagues [8], we re-assayed our Cohort 1 samples with the 3B3/82E1 on the same Erenna platform, with and without addition of Tween 20 (which they had included) (Figure 8). In our hands, the LLoQ for this assay (with Tween 20) was comparable (0.3 pg/ml) with that reported by Savage and colleagues (0.18 pg/ml) [8]. In the Cohort 1 CSF samples, the 3B3/82E1 assay gave oAβ levels ranging from 0.56 to 3.93 pg/ml, and there was no difference between AD (mean 1.8 pg/ml) and controls (mean 1.8 pg/ml). When Tween 20 was added to aliquots of the same CSF samples, the 3B3/82E1 assay detected oAβ that ranged from 0.6 to 3.3 pg/ml. Again there was no difference between AD (mean 1.6 pg/ml) and controls (mean 1.8 pg/ml). This result indicates that treatment with Tween 20 did not change oAβ levels in CSF.

Irrespective of whether or not Tween 20 was added to samples, the 3B3/82E1 assay in our hands yielded values approximately 2.67 times higher than those detected by our 1C22/3D6 assay (mean oAβ: 0.9 pg/ml in AD group and 0.7 pg/ml in controls). Importantly, direct comparison of the values obtained with the 3B3/82E1 assays versus those obtained with 1C22/3D6 revealed little correlation between the two assays, a finding that suggests the assays are not detecting precisely the same species. Nevertheless, none of the three assay formats reveal a significant difference between AD and control subjects.

### Intra-assay and inter-assay precision

To examine intra-assay precision, ADDLs standards ranging from 10 to 0.0094 pg/ml were reversed and loaded in triplicate as samples and analyzed using the same ADDL standards. Above the 0.0781 pg/ml concentration of ADDLs, after calculating by averaging the results of the triplicates and calculating the CV percentage, the CVs averaged 11% and ranged between 3 and 18%. The recovery averaged 107% and ranged between 97 and 114%.

Inter-assay precision was controlled by running one CSF sample in triplicate on each of six different plates. Inter-assay precision calculated by averaging results of the six experiments, and the resultant CV percentage was 11% with a range between 3% and 28%.

## Discussion

Our results describe an Erenna-based immunoassay, using mAb 1C22 for capture and fluorescently-labeled mAb 3D6 for detection, which can selectively and sensitively quantify synthetic and natural soluble oAβ of a wide size range. Using this new o-ELISA, we were able to quantify signals from biological samples, including soluble extracts of AD brain and human CSF.

We chose this immunoassay design in an attempt to confirm and extend the recently described Erenna-based o-ELISAs developed by Esparza and colleagues [9] and Savage and colleagues [8]. First, Esparza and colleagues bound their N-terminal capture antibody HJ3.4 to microtiter plates (rather than beads) and used this same antibody for detection, achieving a LOD of 1.56 pg/ml and a LLoQ of 6.25 pg/ml [9]. With these sensitivities, they could detect oligomers in human brain but not in CSF. Next, Savage and colleagues immobilized their capture antibody (19.3, a humanized antibody with the same complementary determining regions as 3B3) on magnetic beads and detected with mAb 82E1 (an N-terminal specific mAb, like 3D6). The signal-to-noise ratio improved with this bead-based assay, achieving a LOD of 0.09 pg/ml and a LLOQ of 0.18 pg/ml, and oligomers were detected in most CSF samples analyzed [8].

Here, we compared our oAβ-preferring capture antibody, 1C22, with the murine version of 19.3 (that is, 3B3), combining each with 3D6 or 82E1, initially on the MSD platform. 1C22/3D6 allowed the best sensitivity, achieving: LOD of 4.0 pg/ml and LLoQ of 18.7 pg/ml when using ADDLs as the standard; LOD of 55.6 pg/ml and LLoQ of 187 pg/ml using (S26C)_2_ as the standard; and LOD of 154 pg/ml and LLoQ of 601 pg/ml using DiY as the standard. In these MSD platform assays, 1C22/3D6B was more sensitive than 3B3/3D6B, 1C22/82E1 and 3B3/82E1. We used the best pair, 1C22/3D6, to create a bead-based Erenna assay.

Based on the LLoQs achieved with each of the three synthetic Aβ standards, we chose ADDLs as the optimal standard for quantifying a broad range of oligomer sizes. We hypothesize that the greater assay sensitivity achieved using ADDLs as a standard occurs because the ADDL mixture contains more high-molecular-weight oligomers than the DiY or (S26C)_2_ preparations. This provides more potential binding sites and thus greater antibody avidity, resulting in tighter binding and greater sensitivity to detect small quantities of oligomers.

Having selected the best antibody pair and the optimal synthetic standard for a bead-based Erenna o-ELISA, we continued to validate its selectivity and specificity. First, this o-ELISA was shown to have 26,000-fold selectivity for synthetic oligomers over a synthetic Aβ40 monomer preparation that was shown to lack oligomers. Consistent with prior reports [3,22-25], the monomer concentrations in our CSF samples were always less than 10 ng/ml. Thus, even in the worst-case scenario, Aβ monomer could only produce a false-positive oligomer signal of ≤0.47 pg/ml and would by definition produce the highest interference in samples with the highest monomer levels. However, we observed no significant correlation between Aβ42 levels and Aβ oligomer levels in any of the three CSF cohorts studied (Additional file 3). These results indicate that the detected oAβ values are not substantially influenced by cross-reactivity with Aβ monomers. Second, ADDLs were fractionated by SEC using a Superdex 200 column and the fractions used for o-ELISA. The results indicate that the o-ELISA recognizes a broad range of oAβ, but is particularly suited to detect assemblies of masses between ~40 and 670 kDa (that is, within the included volume of a Superdex 200 column). Moreover, the lack of reaction with SEC-isolated Aβ42 monomer further confirms the selectivity of the o-ELISA for oAβ. Third, the o-ELISAs specifically detected natural oAβ in soluble human brain extracts and CSF samples. Quantitative immunodepletion of Aβ (that is, IPs with 1282 and AW7) from the AD-TBS brain extracts or from CSF (each confirmed by western blotting) reduced the o-ELISA signals by 98 to 100% of their original values. The 1C22/3D6 oligomer signal thus arises from oAβ in biological samples.

We then performed initial experiments to ask whether this o-ELISA could be used for quantifying oligomeric Aβ forms in the CSF as an AD biomarker by analyzing three different sets of patient samples obtained from different clinical centers. In two of these cohorts (Cohorts 1 and 2), CSF samples of clinically diagnosed AD patients showed no significant difference in oAβ levels compared with controls. However, there was a small but significant difference between AD and MCI patients in Cohort 2 from the Mayo Clinic CSF collection. In the third cohort, from the Harvard Biomarker Study collection, clinically diagnosed MCI patients also showed a small but significant increase in mean oligomer levels compared with age-matched controls. The three well-established CSF biomarkers for AD – levels of tau, phosphorylated tau and Aβ42 – distinguished control and AD patients much better than did soluble oAβ levels determined by the current o-ELISA.

AD patients might be expected to have increased concentrations of oAβ in CSF since the levels would reflect the high levels of aggregated Aβ accumulating in the brain parenchyma in the form of amyloid deposits. It is possible that patients in the early clinical stages of AD (for example, MCI) may have somewhat higher levels of soluble oAβ in their CSF compared with patients in later stages. We found in Cohorts 2 and 3 that patients with MCI had increased mean levels of CSF oligomers compared with control and AD subjects. Soluble oAβ may thus increase as these species begin to accumulate in brain parenchyma and populate what initially are mostly diffuse plaques. However, the substantial overlap of oligomer levels among control, MCI and AD groups in the three available cohorts we analyzed indicates that, despite similar sensitivities of our Erenna immunoassays, we did not observe the significant differences between AD and control subjects reported in the two cohorts studied by Savage and colleagues [8]. We also did not find a significant correlation between MMSE and oAβ levels in this limited number of subjects. Direct comparison of the 3B3/82E1 antibody pair (Additional file 4), which is very closely similar to that used in Savage and colleagues’ o-ELISA, with our own antibody pair (1C22/3D6) on Cohort 1 CSF samples showed that 3B3/82E1 gave about 2.67 times higher Aβ oligomer values but with the same outcome – that is, no significant difference between AD and control subjects. One explanation for the higher apparent Aβ oligomer levels using 3B3/82E1 may be greater monomer cross-reactivity. The calculated cross-reactivity of 3B3/82E1 was reported by Savage and colleagues to be between 0.03 and 0.05%, so if we assume that the highest level of Aβ monomer in CSF is ~10 ng/ml, the 3B3/82E1 assay could have an oAβ signal emanating from monomers of about 4 pg/ml, compared with just 0.4 pg/ml calculated for our 1C22/3D6 assay.

Savage and colleagues studied CSF samples from two cohorts; that is, a total of 52 AD subjects and 43 controls. oAβ levels ranged from 0.75 to 3.3 pg/ml in AD subjects and from undetectable to 0.66 pg/ml in controls. On average, their Aβ40 values were 1,982 pg/ml for the AD group and 2,208 pg/ml for controls, and their mean Aβ42 levels for AD and control were 98 and 188 pg/ml, respectively. Across our three cohorts (from a total of 80 subjects), we found oAβ values ranged from undetectable to 2.42 pg/ml for AD, from undetectable to 2.74 pg/ml for MCI, and from undetectable to 1.46 pg/ml for controls, with the Aβ42 levels significantly lower in AD and MCI than controls.

Because of the important role that soluble oAβ is believed to have in AD pathogenesis, interest in the specific detection of oligomers in CSF has gained much attention. Five prior studies reported higher mean levels of oAβ in CSF from AD versus controls, but all found considerable overlap of the values. Fukumoto and colleagues [26] and Savage and colleagues [8] reported a significant difference of mean oligomer levels in AD versus control groups, and an inverse correlation with the MMSE scores of the patients. Holtta and colleagues [5] and Herkovits and colleagues [6] reported that oligomer levels differed between AD and controls but were less effective at discriminating AD from controls than Aβ^42^ or t-tau and P-tau181, and they reported no statistical correlation between oAβ levels and MMSE scores. Of note, Holtta and colleagues found that AD patients with mild to moderate AD had significantly higher levels of oAβ than controls, but patients with severe AD did not differ significantly from controls, potentially similar to our MCI findings here.

Taken together, current evidence suggests that soluble oligomers can be detected in human CSF, but that oAβ as currently measured are not yet a useful clinical biomarker. However, the study of oAβ is still at an early stage. Only a handful of o-ELISAs have been thoroughly validated and the conditions employed for CSF collection, processing and storage and how these may affect oAβ detection have yet to be investigated. Perhaps more importantly, little effort has been made to develop assays that detect specific populations of oAβ. It is therefore crucial to develop even more specific assays to measure different oligomer subgroups in a manner analogous to how we detect different monomer forms such as Aβ40 and Aβ42. This may require more specific capture and detection antibodies and perhaps use even more sensitive amplification platforms than employed up to now. Given the potential utility of oAβ as theragnostic markers for testing anti-amyloid therapeutics, a continued effort to better understand and accurately measure different oligomeric forms of Aβ is warranted.

## Conclusions

oAβ are present in most samples of human CSF, but the levels are very low and their reliable detection requires the use of high-sensitivity platforms. Oligomer levels detected by the current assays tend to be higher in CSF from subjects with MCI than in AD or age-matched control subjects, but there was considerable overlap between the levels found in all three groups, whereas the accepted AD CSF biomarkers Aβ42, tau and p-tau clearly distinguished AD and MCI from controls. Further work, including the development of assays that detect specific populations of oAβ, is required to determine whether measurement of oAβ has diagnostic or theragnostic potential.

## Additional files

Additional file 1: Figure S1. Showing SEC and electron microscopy (EM) analysis of ADDLs. A portion of the ADDL o-ELISA standard was chromatographed on a Superdex 75 5/150 analytical size exclusion column eluted with PBS, pH 7.4, and analyzed by negative contrast EM (inset). The elution of Aβ is shown in blue and the chromatogram produced by the Ham’s F12/dimethylsulfoxide (DMSO) vehicle is shown in red. EM of ADDLs (left) and the Ham’s F12/DMSO vehicle (right). Additional file 2: Figure S2. Showing SEC isolation of WT, DiY and (S26C)_2_. Aβ monomer (A) and dimers (C and E) were isolated using a Superdex 75 10/300 size exclusion column eluted with 50 mM ammonium bicarbonate, pH 8.5. The concentration of monomer and dimers was determined by absorbance and samples were diluted as required, and aliquots flash frozen in liquid nitrogen and stored at −80°C until use. Once thawed a portion of each (B, WT; D, DiY; and F, (S26C)_2_) was analyzed for the presence of soluble aggregates using a Superdex 200 3.2/300 analytical size exclusion column. The elution of globular protein standards is indicated by black arrows at the top of each chromatogram. The red arrow in (F) indicates the presence of a small amount of high molecular weight aggregates. Additional file 3: Figure S3. Showing that Aβ42 and oAβ levels present no significant correlation in any of the three cohorts studied. The oligomer values measured using the 1C22/3D6 o-ELISA and the Aβ42 values obtained using standard immunoassays are shown for Cohort 1 (A), Cohort 2 (B) and Cohort 3 (C). Oligomer and Aβ42 values were analyzed using Pearson correlation, *P* >0.5, two-tailed. Additional file 4: Figure S4. Showing that the 3B3/82E1 o-ELISA on the Erenna platform specifically recognizes oAβ, not monomers, and detects human Aβ species in AD-TBS brain extracts. (A) Aβ monomers show virtually no reactivity in the 3B3/82E1 o-ELISA. (B) Soluble extracts of AD brains was serially diluted and assayed with the Erenna 3B3/82E1 o-ELISA, revealing highly linear concentration curves in the brain extract. SAT, o-ELISA value of the highest loaded standard (50 pg/ml).

## Abbreviations

Aβ: amyloid beta
(Aβ(1–40))_DiY_: dityrosine cross-linked amyloid-beta dimer
(Aβ(1–40)S26C)_2_: cystine cross-linked amyloid-beta dimer
AD: Alzheimer’s disease
ADDL: amyloid-beta-derived diffusible ligand
B: biotinylated
CSF: cerebrospinal fluid
CV: coefficient of variance
DiY: dityrosine
LLoQ: lower limit of reliable quantification
LOD: limit of detection
mAb: monoclonal antibody
MCI: mild cognitive impairment
MMSE: Mini-Mental State Examination
MP: microparticle
oAβ: amyloid-beta oligomers
o-ELISA: oligomer-specific enzyme-linked immunosorbent assay
p-tau: phosphorylated tau
SEC: size exclusion chromatography
TBS: Tris-buffered saline
t-tau: total tau
