# Supplementary figures and images for: A highly sensitive novel immunoassay specifically detects low levels of soluble Aβ oligomers in human cerebrospinal fluid
Source: Alzheimers Res Ther. 2015 Mar 22;7(1):14. doi: 10.1186/s13195-015-0100-y (PMC4369838; doi:10.1186/s13195-015-0100-y)

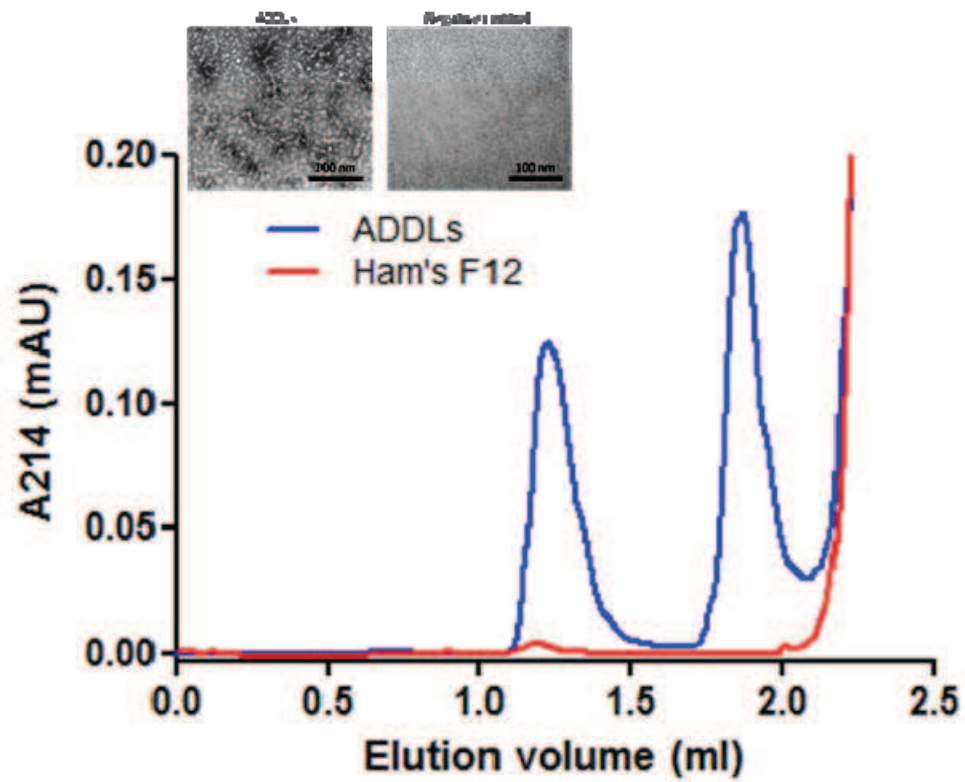

Supplementary Figure 1 SEC and EM analysis of ADDLs

Supplement: Additional file 1: Figure S1. — Showing SEC and electron microscopy (EM) analysis of ADDLs. A portion of the ADDL o-ELISA standard was chromatographed on a Superdex 75 5/150 analytical size exclusion column eluted with PBS, pH 7.4, and analyzed by negative contrast EM (inset). The elution of Aβ is shown in blue and the chromatogram produced by the Ham’s F12/dimethylsulfoxide (DMSO) vehicle is shown in red. EM of ADDLs (left) and the Ham’s F12/DMSO vehicle (right). [file 13195_2015_100_MOESM1_ESM.pdf]

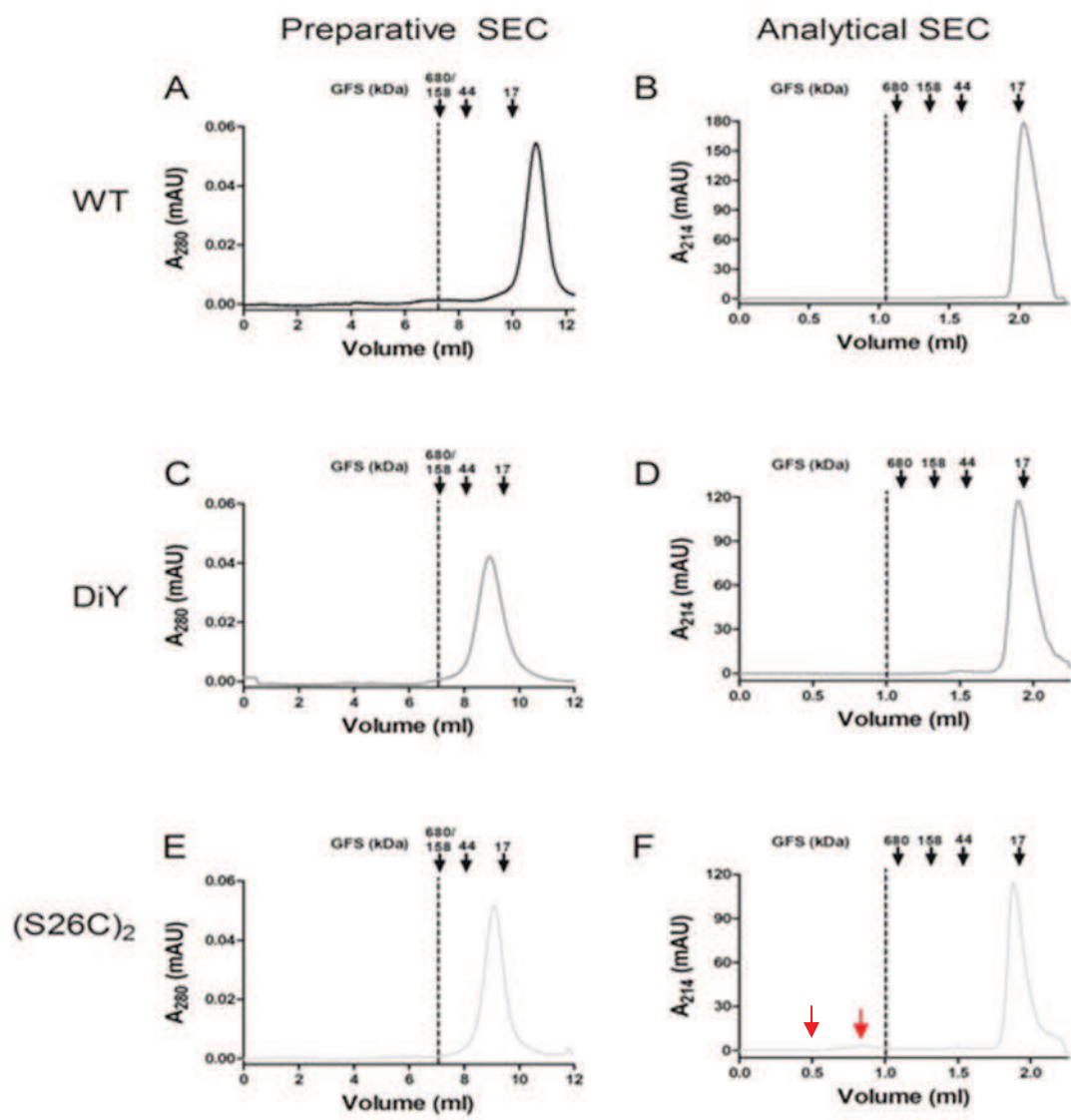

Supplementary Figure 2 SEC isolation of WT, DiY and [S26C]<sub>2</sub>

Supplement: Additional file 2: Figure S2. — Showing SEC isolation of WT, DiY and (S26C)2. Aβ monomer (A) and dimers (C and E) were isolated using a Superdex 75 10/300 size exclusion column eluted with 50 mM ammonium bicarbonate, pH 8.5. The concentration of monomer and dimers was determined by absorbance and samples were diluted as required, and aliquots flash frozen in liquid nitrogen and stored at −80°C until use. Once thawed a portion of each (B, WT; D, DiY; and F, (S26C)2) was analyzed for the presence of soluble aggregates using a Superdex 200 3.2/300 analytical size exclusion column. The elution of globular protein standards is indicated by black arrows at the top of each chromatogram. The red arrow in (F) indicates the presence of a small amount of high molecular weight aggregates. [file 13195_2015_100_MOESM2_ESM.pdf]
