# Supplementary material for: A highly sensitive novel immunoassay specifically detects low levels of soluble Aβ oligomers in human cerebrospinal fluid
Source: Alzheimers Res Ther. 2015 Mar 22;7(1):14. doi: 10.1186/s13195-015-0100-y (PMC4369838; doi:10.1186/s13195-015-0100-y)
Supplement: Additional file 3: Figure S3. — Showing that Aβ42 and oAβ levels present no significant correlation in any of the three cohorts studied. The oligomer values measured using the 1C22/3D6 o-ELISA and the Aβ42 values obtained using standard immunoassays are shown for Cohort 1 (A), Cohort 2 (B) and Cohort 3 (C). Oligomer and Aβ42 values were analyzed using Pearson correlation, P >0.5, two-tailed. [file 13195_2015_100_MOESM3_ESM.pdf]

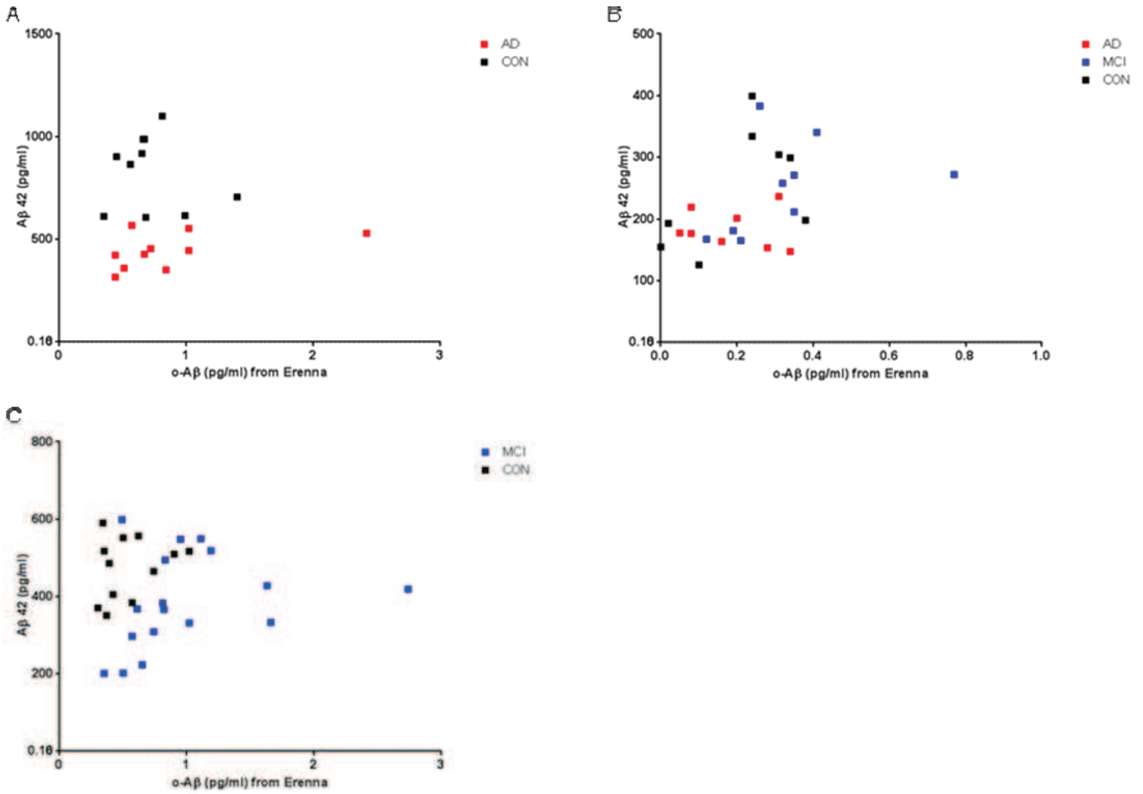

Supplementary Figure 3 Aβ42 and Aβ oligomers levels show no significant correlation in any of the three cohorts studied.
