# Supplementary material for: A highly sensitive novel immunoassay specifically detects low levels of soluble Aβ oligomers in human cerebrospinal fluid
Source: Alzheimers Res Ther. 2015 Mar 22;7(1):14. doi: 10.1186/s13195-015-0100-y (PMC4369838; doi:10.1186/s13195-015-0100-y)
Supplement: Additional file 4: Figure S4. — Showing that the 3B3/82E1 o-ELISA on the Erenna platform specifically recognizes oAβ, not monomers, and detects human Aβ species in AD-TBS brain extracts. (A) Aβ monomers show virtually no reactivity in the 3B3/82E1 o-ELISA. (B) Soluble extracts of AD brains was serially diluted and assayed with the Erenna 3B3/82E1 o-ELISA, revealing highly linear concentration curves in the brain extract. SAT, o-ELISA value of the highest loaded standard (50 pg/ml). [file 13195_2015_100_MOESM4_ESM.pdf]

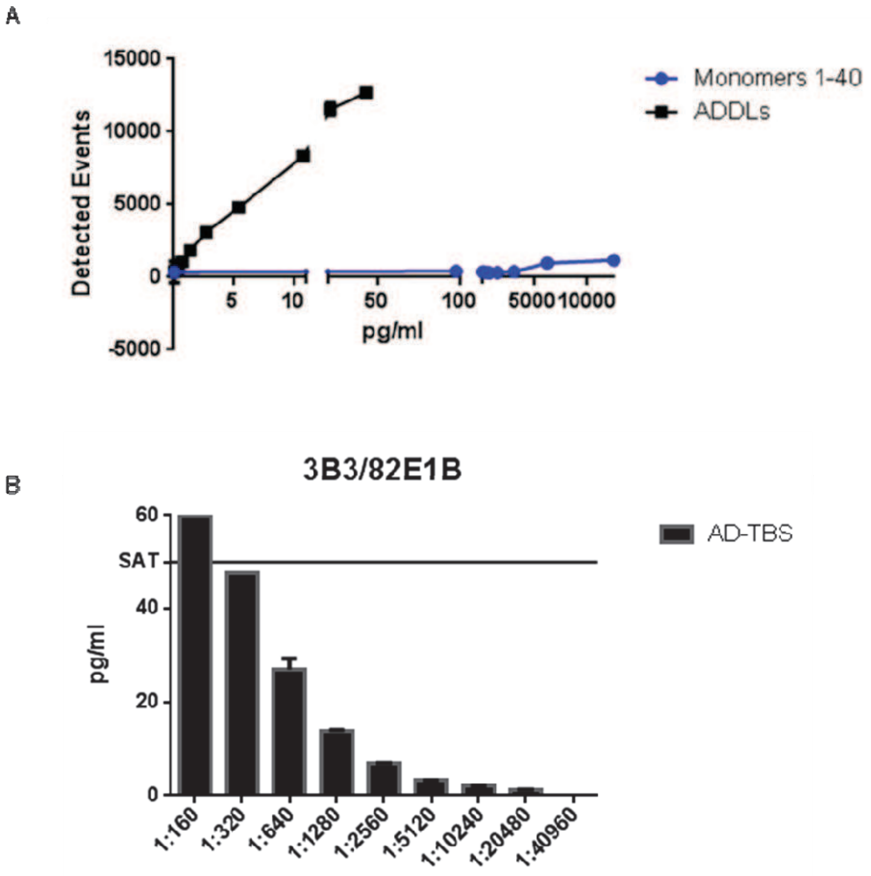

Supplementary Figure 4 The 3B3/82E1 o-ELISA on the Erenna platform specifically recognizes Aβ oligomers, not monomers, and detects human Aβ species in AD-TBS brain extracts.
